# Biotransformation Is an Effective Mechanism for Modulating the Biological Toxicity of Nodularin (NODR)

**DOI:** 10.3390/toxins18020091

**Published:** 2026-02-11

**Authors:** Chunyu Fu, Mengchen Li, Qiannan Shi, Yixue Xu, Wansong Zong

**Affiliations:** 1 College of Geography and Environment, Shandong Normal University, 88# East Wenhua Road, Jinan 250014, China; 2 Medical and Health College, Binzhou College of Science and Technology, 888# Bohai 24th Road, Binzhou 256606, China

**Keywords:** nodularin-R, protein phosphatase 1, molecular simulation, biotransformation pathway

## Abstract

The biotransformation of nodularin (NOD) is one of the critical strategies for regulating their biological toxicity. To investigate the effects and mechanisms of the biotransformation pathway, this study synthesized six biotransformation products of nodulein-R (NODR-BTPs) and evaluated their inhibitory effects on protein phosphatase 1 (PP1) through protein phosphatase inhibition assays. The inhibitory effects of NODR-BTPs diminished as the molecular weight and polarity of the introduced biological thiols increased, indicating that biotransformation is an efficient mechanism for modulating the biological toxicity of NODR. Through ligand replacement and molecular docking techniques, the potential regulatory mechanisms underlying the primary interaction processes between NODR-BTPs and PP1 were further elucidated. The introduced biological thiols improved the hydrogen bonding for Glu_275_ ← “Mdhb^5^”and enhanced the electropositive–electronegative interactions between “Mdhb^5^” and PP1. This resulted in an increase in the positive accessible surface area, negative accessible surface area, and polar surface area at the interface of “Mdhb^5^” and PP1. The biothiol moiety subsequently enhanced hydrogen bonds for Arg_96_ → MeAsp^1^ and Arg_96_ → Glu^4^, thereby affecting the binding of these key interaction sites to PP1. This further diminished interactions between conserved amino acids in PP1 and Mn^2+^ ions, including the ionic bond for Asp_92_-Mn_1_^2+^ and metal bonds for Asp_64_-Mn_1_^2+^ and His_66_-Mn_1_^2+^, leading to increased exposure of Mn^2+^ ions. The regulatory mechanisms facilitated the restoration of PP1 catalytic activity.

## 1. Introduction

Nodularin (NOD) is a cyclic pentapeptide hepatotoxic toxin produced by *Nodularia spumigena*, typically found in eutrophic brackish or semi-brackish water bodies such as the Baltic Sea [1]. When cyanobacteria proliferate extensively and form blooms, the water may contain high concentrations of NOD. The primary target organ of nodularin is the liver, and its mechanism of toxicity is similar to that of another well-known cyanobacterial toxin—microcystin (MC) [2]. NOD is actively and efficiently taken up into hepatocytes via organic anion transporting polypeptides (OATPs) on the hepatocellular membrane [3]. Upon entering the cell, nodularin potently and irreversibly inhibits the activity of protein phosphatases (PPs), specifically protein phosphatase 1 (PP1) and protein phosphatase 2A (PP2A) [4,5]. PPs are key enzymes responsible for maintaining the balance of intracellular protein phosphorylation. PP1 and PP2A belong to the protein phosphatases superfamily and are responsible for over 90% of phosphatase reactions in eukaryotes. As a major serine/threonine phosphatase, PP1 is highly conserved and ubiquitously expressed across all eukaryotes. It regulates numerous critical cellular processes, including meiosis, cell cycle progression, protein synthesis, apoptosis, dynamics, cytoskeletal and glycogen metabolism [4]. Their inhibition by nodularin leads to the hyperphosphorylation of cellular proteins. This, in turn, disrupts cytoskeletal structures (such as keratins and microfilaments), interferes with signal transduction, and induces oxidative stress, ultimately resulting in hepatolysis, necrosis, and massive hemorrhage [5,6,7]. NOD can accumulate in human tissues through three primary exposure routes: ingestion of cyanobacterial cells, consumption of contaminated food, or direct uptake of dissolved toxins from water [5,8,9]. Chronic exposure to NOD is associated with an increased risk of cancer.

Since the initial discovery of NOD, ten variants have been identified to date [6,10]. Among them, Nodularin-R (NODR) is the most extensively studied variant [11]. The structure of NODR consists of 3-amino-9-methoxy-2,6,8-trimethyl-10-phenyl-4,6-decadienoic acid (Adda), D-glutamic acid (D-Glu), N-methyldehydrobutyric acid amide (Mdhb), D-erythro-β-methylaspartic acid (D-MeAsp) and L-arginine (L-Arg) [12,13]. MC is a monocyclic heptapeptide with pronounced hepatotoxicity. Its primary structure is -D-Ala-L-X-D-MeAsp-L-Z-Adda-D-Glu-Mdha, where X and Z denote variable amino acids [14]. NOD and MC exhibit a high degree of similarity [15]. From a structural perspective, both are cyclic peptides containing the Adda moiety, which is essential for their toxicity. In the NOD structure, the Mdhb residue at position 7 contains an unsaturated bond, while in the MC structure, the Mdha residue at position 5 also contains an unsaturated bond [16]. Regarding their mechanism of toxicity, both are specifically transported into hepatocytes via OATP carriers and exert their effects by inhibiting protein phosphatases PP1 and PP2A, leading to liver damage and tumor promotion [13,17]. Ecologically and in terms of health impacts, they are both products of eutrophic water bodies and pose threats to ecosystems and human health through similar pathways. Due to their shared chemical properties, NOD and MC are typically studied using the same analytical methods [18].

Given the hepatotoxicity of NOD, it is important to control their environmental risks. Currently, known water treatment strategies include biological degradation and chemical degradation [19,20]. In addition to the methods mentioned above, biotransformation pathways are considered a promising strategy for regulating the toxicity of NOD [21]. Previous studies have shown that NOD can conjugate with intracellular glutathione (GSH) to form NOD-GSH. The toxicity of this conjugate is markedly reduced compared to that of the NOD, which may represent the first stage of detoxification [22]. Furthermore, previous studies have demonstrated the feasibility of regulating the inhibitory effects of MC on PPs through biotransformation pathways. The unsaturated Mdha^7^ in MC can form covalent bonds with free biological thiols, resulting in the formation of MC biotransformation products (MC-BTPs) [23,24,25]. Given the high structural and toxicological similarity between NOD and MC, free biological thiols are expected to react with the unsaturated Mdhb^5^ in NODR, forming electrophilic reaction products termed biotransformation products of nodularin-R (NODR-BTPs) [6,17,24].

In order to evaluate the regulatory effect of biotransformation pathways on NODR toxicity, the first issue that needs to be addressed is the preparation of sufficient NODR-BTPs. Since NODR-BTPs are electrophilic conjugation products of NODR with biological thiols, their synthesis in sufficient quantities can be achieved through in vitro simulation of the electrophilic addition reactions between NODR and thiol-containing biomolecules [25]. Subsequently, following standard isolation and purification protocols for NODR, NODR-BTPs were further purified and prepared for subsequent toxicity assessments. However, the elucidation of molecular mechanisms underlying biotransformation pathway regulation of NODR toxicity remains challenging due to the unavailability of crystallographic structures for the NODR-BTPs-PP1 complex [26]. Inspired by previous studies, this research employed a homology modeling strategy and molecular docking simulations to construct a model of the NODR-BTPs-PP1 complex, thereby clarifying the elucidation of their interaction mechanisms at a molecular level [27,28].

**Figure 1:**
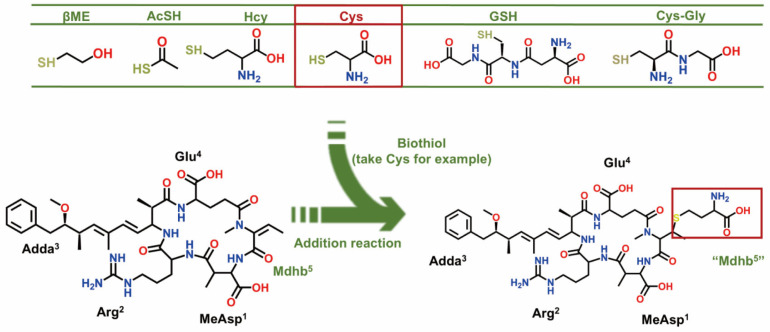
Schematic diagram of BTPs resulting from the nucleophilic addition reaction of biological thiols to Mdhb^5^.

In this study, six representative NODR-BTPs were synthesized via electrophilic addition reactions between NODR and biological thiols (Figure 1), followed by identification using liquid chromatography (LC) and mass spectrometry (MS). The inhibitory effects of NODR and NODR-BTPs on PP1 were evaluated using conventional PP inhibition assays. Subsequently, molecular docking simulations were employed to construct interaction models of NODR-BTPs-PP1 complexes, based on the established NODR-PP1 complex framework. We integrated the inhibition test data with candidate interaction parameters to identify key factors associated with the biological toxicity of NODR-BTPs, elucidating the molecular mechanism through which the biotransformation pathway modulates NODR toxicity. This study contributes to the refinement of regulatory strategies concerning the biotoxicity of NODR, and is expected to draw attention to the secondary environmental risks posed by NODR-BTPs, thereby helping to minimize their impact on human health. This study not only advances the comprehensive understanding of NOD-BTP toxicity, but also demonstrates the feasibility of modulating NOD toxicity through exogenous biological thiols, thereby providing a potential strategy for toxicity mitigation. These findings therefore hold significant theoretical and practical value.

## 2. Results and Discussion

### 2.1. Identification and Preparation of NODR-BTPs

**Figure 2:**
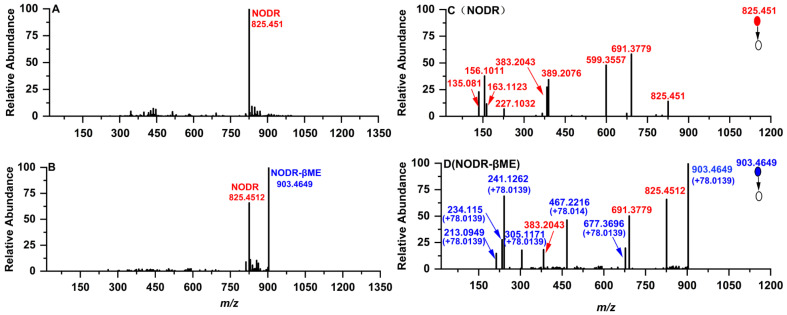
Analysis of NODR and its electrophilic adduct product NODR-βME. Experimental conditions: (**A**) mass spectrum of NODR; (**B**) mass spectrum of NODR-βME; (**C**) MS/MS spectrum of NODR; (**D**) MS/MS spectrum of NODR-βME.

Additional structural characterization of NODR-BTPs was conducted utilizing tandem mass spectrometry. Utilizing tandem mass spectrometry results, the identification of NODR-BTPs was accurately accomplished by analyzing the secondary fragmentation patterns of the prepared NODR-BTPs in comparison to those of NODR. Coupled with biological thiols, NODR was transformed into NODR-BTPs of different molecular masses, and the above conversion products were characterized by high-resolution mass spectrometry (MS error < 2 ppm). For NODR (C_41_H_60_N_8_O_10_), the mass spectral signal at *m*/*z* 825.451 corresponded to its monoprotonated product (Figure 2A). In selected electrophilic adduct samples, NODR remained detectable but exhibited lower signal intensity compared to the newly formed ions, with a prominent signal at *m*/*z* 903.4649. Since the molecular weight of βME was 78.0139 Da, *m*/*z* 903.4649 should be attributed to the conjugation product of βME to NODR (Figure 2B). For other electrophilic adduct samples, five novel monoprotonated NODR-BTPs were detected (Figures S1 and S2C–G). Similarly, based on the molecular weights of AcSH (75.9983 Da), Cys (121.0198 Da), Hcy (135.0354 Da), GSH (307.0838 Da), and Cys-Gly (178.0412 Da), the newly observed MS signals at *m*/*z* 901.4493, 946.4708, 960.4864, 1132.5348, and 1003.4922 were identified as the monoprotonated conjugation products of NODR with AcSH, Cys, Hcy, GSH, and Cys-Gly, respectively.

The structure of NODR-BTPs was further identified by comparing the characteristic fragment ions in the MS/MS spectra of NODR with those of NODR-BTPs. Partial fragment ions of NODR were detected at *m*/*z* 135.0810 ([(-Arg^2^-) + H]^+^), 156.1001 ([-C_11_H_15_O-]^+^), 163.1123 ([(-Glu^4^-Mdhb^5^-) + H]^+^), 227.1032 ([(-Mdhb^5^-MeAsp^1^-Arg^2^-) + H]^+^), 383.2043 ([-C_11_H_15_O-Glu^4^-Mdhb^5^-]^+^), 389.2076 ([(-Arg^2^-Adda^3^-Glu^4^-) + H]^+^), 599.3557 ([(-Arg^2^-Adda^3^-Glu^4^-) + H]^+^), and 691.3779 ([-Glu^4^-Mdhb^5^-MeAsp^1^-Arg^2^-C_11_H_17_NO-] + 2H]^+^), corresponding to secondary structures as shown in Figure 2C. NODR-βME not only shared several fragment ions with NODR (e.g., *m*/*z* 383.2043, 691.3779) but also exhibited novel fragment ions at *m*/*z* 213.0949, 234.1150, 241.1262, 305.1171, 467.2216, and 677.3696 (Figure 2D). These new ions corresponded to ([PhCH_2_CH(OCH_3_)]^+^, *m*/*z* + 78.0139), ([(-Arg^2^-) + H]^+^, *m*/*z*+ 78.0139), ([-C_11_H_15_O-]^+^, *m*/*z* + 78.0139), ([(-Glu^4^-Mdhb^5^-) + H]^+^, *m*/*z*+ 78.0139), ([-C_11_H_15_O-Glu^4^-Mdhb^5^-]^+^, *m*/*z* + 78.0139) and ([(-Mdhb^5^-MeAsp^1^-Arg^2^-) + H]^+^, *m*/*z*+ 78.0139). These new ions are derived from the modification of NODR’s parent fragments by βME conjugation. Using the same analytical strategy, other NODR-BTPs were characterized (Table S1). Comparative analysis of these fragment ions revealed that all novel fragments of NODR-βME exhibit a consistent mass increment of 78.0139 Da (the molecular weight of βME) compared to their corresponding parent fragments in NODR, and this modification is consistently associated with the Mdhb^5^ residue. In summary, we speculated that NODR-BTPs were generated through electrophilic addition reactions between various biological thiol compounds (including βME, Cys, GSH, AcSH, Hcy, and Cys-Gly) and the unsaturated carbonyl group in the Mdhb^5^ residues.

### 2.2. Assessment of the Inhibitory Effects of NODR/NODR-BTPs on PP1

To investigate the regulatory effects of biotransformation pathways, NODR-BTPs were purified from the crude extracts via preparative chromatography. The preparation and purification details of NODR-BTPs are summarized in Table S2. Due to their high concentrations (>1200 µmol/L) and purity (>95.79%), the prepared samples were directly utilized in PP1 inhibition assay.

**Figure 3:**
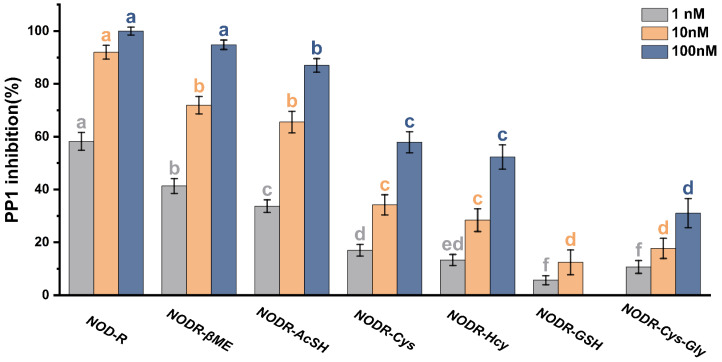
Inhibitory effects of NODR and NODR-BTPs on PP1. Error bars represent the standard error of three replicate analyses. Significant differences between NODR and NODR-BTPs were analyzed using one-way ANOVA followed by the LSD post hoc test. Different lowercase letters denote statistically significant differences (*p* < 0.05) between groups. For example, at the 1 nm level, letters a and b represent significant differences between NODR and NODR-βME; letter f for both NODR-GSH and NODR-Cys-Gly indicate no significant difference between them. Analysis was conducted using SPSS software (version 27.0; IBM Corp., Chicago, IL, USA).

All NODR-BTPs inhibited PP1 activity, but these inhibitions were weaker than those of NODR (Figure 3). The decrease in PP1 activity became more pronounced with increasing toxin concentration, showing a dose-dependent relationship. Statistically significant differences (*p* < 0.05) in PP1 inhibition were observed between the NODR and NODR-BTPs groups. At 1 nM, the inhibitory effects of toxins were categorized into six classes (a: NODR; b: NODR-βME; c: NODR-AcSH; d: NODR-Cys, NODR-Hcy; e: NODR-Hcy; f: NODR-GSH and NODR-Cys-Gly) based on one-way ANOVA followed by the LSD post hoc test. Using the same strategy, the inhibitory effects were classified into four classes at 10 nM and six classes at 100 nM. Overall, the inhibition potency followed the order: NODR > NODR-βME > NODR-AcSH > NODR-Cys > NODR-Hcy > NODR-GSH > NODR-Cys-Gly. Based on the fundamental properties of biological thiols, we found that the inhibitory activity of NODR-BTPs toward PP1 was significantly correlated with their polarity and molecular weight (|R| > 0.941, *p* < 0.05). Therefore, we speculated that the polarity and steric hindrance of the Mdhb^5^ residue may affect the binding of NODR-BTPs to PP1, thereby regulating their toxicity.

Our findings demonstrated that NODR-BTPs exhibit a significantly reduced toxicity compared to the natural toxin NODR, establishing biotransformation as a viable strategy for toxicity mitigation. Nevertheless, all tested NODR-BTPs still retain non-negligible residual toxicity, which is potentially attributable to the polarity and steric hindrance imposed by the Mdhb^5^ residue in their structure. In conclusion, while biotransformation can effectively diminish NODR toxicity, the potential secondary environmental risks of NODR-BTPs warrant further evaluation.

### 2.3. Simulation of NODR-BTP Interaction with PP1 via Molecular Docking

**Figure 4:**
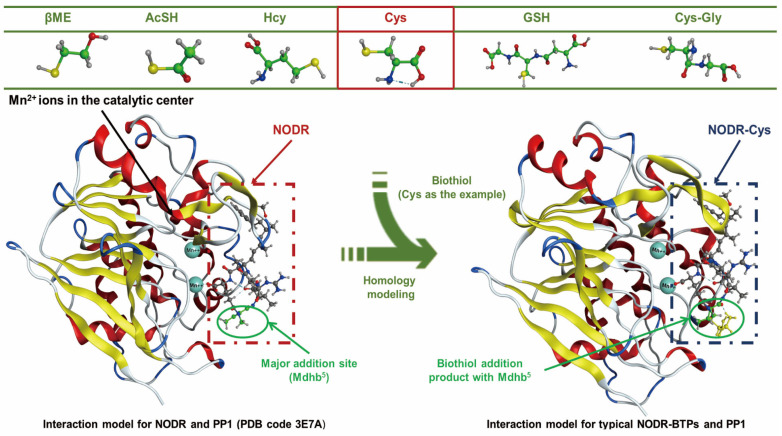
Schematic diagram of the interaction model construction for the NODR-BTP-PP1 complex (with no PDB models) based on ligand replacement strategy.

PP1 inhibition assays showed that biological thiols exert significant regulatory effects on the biotoxicity of NODR. However, due to the lack of the crystal structure of the NODR-BTPs-PP1 complex, the specific regulation mechanism of biological transformation pathway is difficult to study. Based on the structural similarity between NODR-BTPs and the original toxin, we constructed the NODR-BTPs-PP1 complex structure via ligand replacement to elucidate the molecular interactions between them (Figure 4). To further elucidate the molecular basis of these inhibitory differences, we used this molecular docking model to screen 76 candidate interaction parameters between the toxins and PP1, which include positive accessible surface area (ASA^+^), negative accessible surface area (ASA^−^), combination area, polar surface area (ASA^−P^), hydrophobic surface area (ASA^−H^), exposure area associated with the phosphate group, active center exposure, ionic bonds, metal bonds, and hydrogen bonds (see Table S3).

### 2.4. Analysis of Correlation Between Candidate Interaction Parameters and Inhibition Data

**Figure 5:**
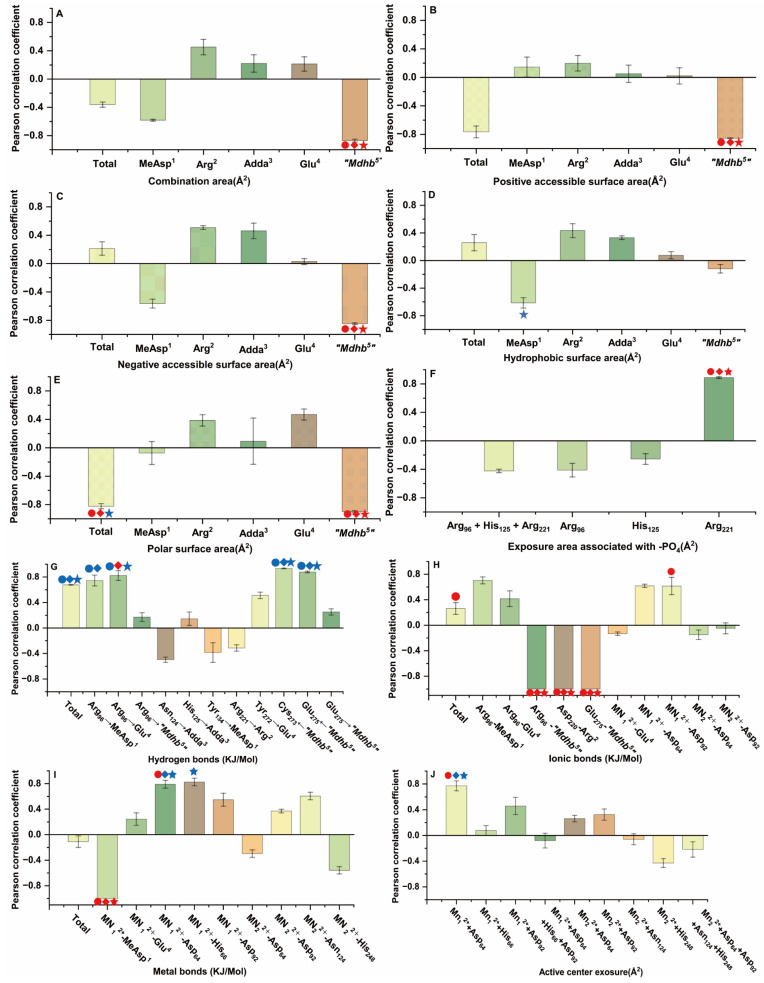
Pearson correlation coefficients between inhibition data and (**A**) combination area, (**B**) positive accessible surface area, (**C**) negative accessible surface area, (**D**) hydrophobic surface area, (**E**) polar surface area, (**F**) exposure area associated with -PO_4_, (**G**) hydrogen bonds, (**H**) ionic bonds, (**I**) metal bonds, and (**J**) active center exposure. Conditions: The symbols 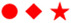 indicate extremely significant correlations (*p* < 0.01) between interaction parameters and inhibition data at 1, 10, and 100 nM toxin concentrations. The symbols 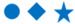 indicate significant correlations (*p* < 0.05) at 1, 10, and 100 nM concentrations.

This study utilized Pearson correlation analysis to evaluate the relationship between inhibition data and candidate interaction parameters. The results indicated notable differences in the correlation between candidate parameters and inhibition data as the toxin concentration varied (Figure 5). At the 1 nM level, 40 candidate interaction parameters showed positive correlations and 36 exhibited negative correlations with the inhibition data, among which six candidate parameters were significantly correlated (*p* < 0.05) and 13 were highly significantly correlated (*p* < 0.01). At the 10 nM level, 42 candidate interaction parameters showed positive correlations and 34 showed negative correlations, of which seven were significantly correlated (*p* < 0.05) and 11 were highly significantly correlated (*p* < 0.01). At the 100 nM level, 44 candidate interaction parameters and 32 were negatively correlated, among which 10 were significantly correlated (*p* < 0.05) and nine were highly significantly correlated (*p* < 0.01). Notably, the number of candidate parameters showing positive correlations with inhibition data increased slightly with increasing toxin concentration, while the number of negatively correlated parameters decreased accordingly. Detailed data are provided in Supplementary Table S4. These results indicate that the correlations between the inhibition data and the candidate interaction parameters vary across different concentration levels. These key interaction parameters are important for exploring the interaction mechanisms between NODR-BTPs and PP1, thereby helping to clarify the molecular mechanisms underlying biotransformation pathways.

**Figure 6:**
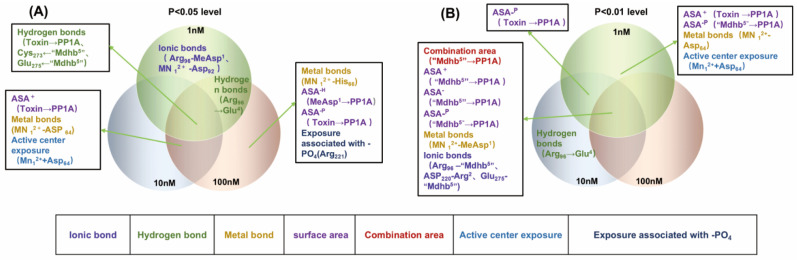
Venn diagrams of significant interaction parameters at the (**A**) *p* < 0.05 level and (**B**) *p* < 0.01 level. Definitions: ASA^+^ denotes the positive accessible surface area; ASA^−^ denotes negative accessible surface area; ASA^−H^ denotes the hydrophobic surface area; ASA^−P^ denotes the polar surface area.

To clarify the diversity of correlations, we employed a Venn diagram to identify key interaction parameters, i.e., those exhibiting significant (*p* < 0.05) or extremely significant (*p* < 0.01) correlations with inhibition data across the three tested concentration levels (1 nM, 10 nM, 100 nM) in Pearson’s correlation analysis. At a significance level of *p* < 0.01(Figure 6B), the following parameters exhibited extremely significant correlations with inhibition data across three toxin concentrations: combination area for “Mdhb^5^” → PP1, positive accessible surface area for “Mdhb^5^” → PP1, negative accessible surface area for “Mdhb^5^” → PP1, polar surface area for “Mdhb^5^” → PP1, metal bonds for Mn_1_^2+^-MeAsp^1^, ionic bonds for Arg_96_-“Mdhb^5^”, Asp_220_-Arg^2^*,* and Glu_275_-“Mdhb^5^”, Arg_221_ and the exposed area of the phosphate group. Additionally, the following parameters showed extremely significant correlations with inhibition data at 1 nM toxin concentration: positive accessible surface area for Toxin → PP1, polar surface area for “Mdhb^5^” → PP1, metal bonds for Mn_1_^2+^-Asp_64_, active center exposure for Mn_1_^2+^+Asp_64_. The polar surface area for Toxin → PP1 showed extremely significant correlations with inhibition data at both 1 nM and 10 nM toxin concentrations.

At the significance level of *p <* 0.05 (Figure 6A), the following interaction parameters showed significant correlations with inhibitory data across toxin concentrations and hydrogen bonds: Toxin → PP1, “Mdhb^5^” → Cys_273_, and “Mdhb^5^” → Glu_275_ correlated significantly with inhibitory data at all three toxin concentrations. Hydrogen bonds for Arg_96_ → Glu^4^ showed significant correlations at 1 nM and 100 nM toxin concentrations. Positive accessible surface area for Toxin → PP1 showed significant correlations at 10 nM and 100 nM toxin concentrations. Metal bonds for Mn^2+^-Asp_64_ and active center exposure (Mn^2+^-Asp_64_) correlated significantly at 10 nM and 100 nM toxin concentrations. Metal bonds for Mn^2+^-His_66_ showed significant correlations at 100 nM toxin concentration. Ionic bonds (Arg_96_-MeAsp^1^ and Mn^2+^-Asp_92_) showed significant correlations at 1 nM toxin concentration. Hydrophobic surface area (MeAsp^1^ → PP1) correlated significantly at 100 nM toxin concentration. Polar surface area (Toxin → PP1) showed significant correlations at 100 nM toxin concentration. Based on the above, interaction parameters that exhibit significant correlations across two or three toxin concentrations may be helpful in elucidating the molecular mechanisms by which biotransformation pathways modulate NODR toxicity.

**Figure 7:**
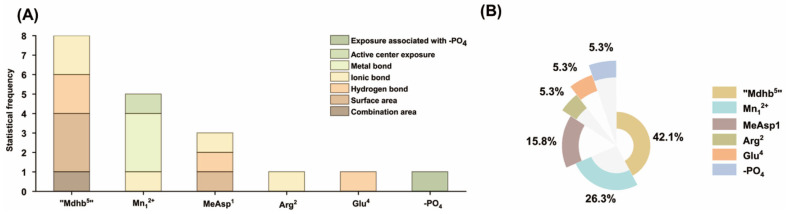
(**A**) Histogram of statistical frequencies associated with key interaction sites and (**B**) pie chart summarizing the total |R| values linked to these sites.

To further clarify the contribution of each functional site to binding, statistical analysis of the aforementioned key interaction parameters was performed to identify critical interaction sites between NODR/NODR-BTPs and PP1. Based on the structures of the toxins, the catalytic center Mn^2+^, and the introduced phosphate group, statistical analysis of key interaction parameters identified critical interaction sites. The statistical frequency histogram showed that eight key interaction parameters were associated with the “Mdhb^5^” residue, five with “Mn_1_^2+^”, three with MeAsp^1^, and one with Arg^2^/Glu^4^/-PO_4_ (Figure 7A). Consistent with the frequency distribution, statistical evaluation of the total mean absolute correlation coefficient (|$\overset{-}{R}$|) for these interaction sites showed that sites participate in the binding of NODR/NODR-BTPs to PP1, with contributions ranked in the following order: “Mdhb^5^” > Mn_1_^2 +^ > MeAsp^1^ > Arg^2^ > Glu^4^ > -PO_4_ (Figure 7B). Specifically, Mn_1_^2+^ and “Mdhb^5^” exerted significant impacts on NODR/NODR-BTPs-PP1 binding. MeAsp^1^ showed considerable influence on the binding. Arg^2^, Glu^4^, -PO_4_ showed moderate effects on the interaction.

The catalytic center Mn^2+^ is important for the catalytic activity of PPs. Key interactions involving Mn^2+^ in the biotransformation pathway play a critical role in restoring the catalytic activity of PP1. Previous studies on nodularin variants have shown that toxins impair PP1 catalytic activity primarily by disrupting the coordination of Mn^2+^ in the active center via direct metal bond interactions [29]. Furthermore, within the structural units of NODR/NODR-BTPs, the “Mdhb^5^” exhibited a greater number of key interaction parameters compared to other sites. This suggests that alterations at the “Mdhb^5^” site may play an important role in regulating NODR toxicity through biotransformation pathways.

### 2.5. Molecular Mechanism of Biological Thiols in Regulating NODR-BTPs and Inhibiting PP1

**Figure 8:**
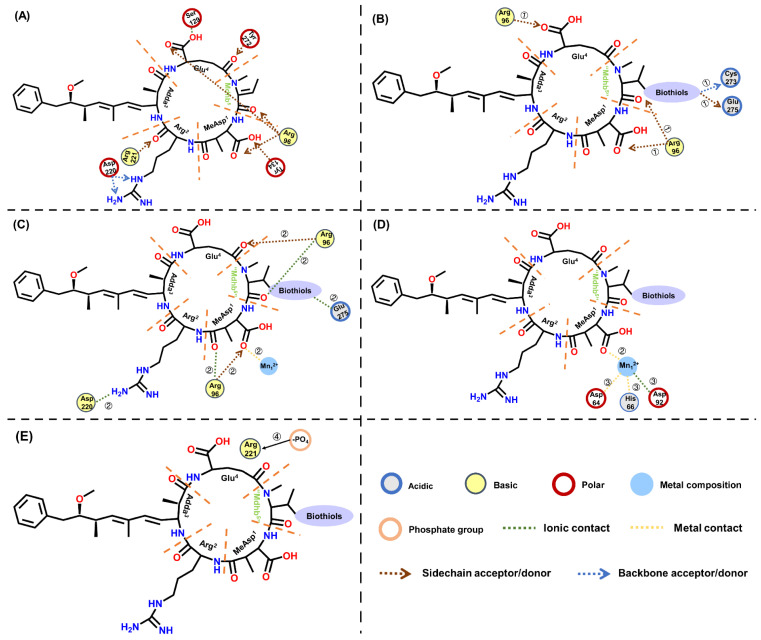
The two-dimensional ligand–receptor interaction diagram for the combination of the toxins to PP1. (**A**) Before the introduction of biological thiols, the direct effect of NODR on PP1; (**B**) the direct effect of the introduced biological thiols on the interactions between the toxins and PP1; (**C**) the effect of the changed “Mdhb^5^” on the interactions between the toxins and the PP1; (**D**) the effect of weak binding between toxins and PP1 on Mn^2+^ interactions; (**E**) The exposure of amino acids bound to phosphate groups was influenced by changes in interactions. Legend explanation: Based on whether the side chains of amino acids carry a charge, they can be classified into polar, acidic, and basic amino acids. In the diagram, these three types are represented by different colors; both side chain acceptors/donors and back bone acceptors/donors refer to hydrogen bonds.

A study by Zong W et al. [25] revealed that glutathione (GSH) conjugation serves as a biotransformation pathway regulating the toxicity of MCs toward protein phosphatase, with toxicity modulation achieved through enhanced hydrogen bonding and covalent interactions. For NODR, however, the regulatory mechanism differs. Notably, NODR does not bind covalently to PP1; instead, as previously reported by Kelker MS et al. [30], it anchors to the active site of PP1 via extensive hydrogen bonding. Based on this, this study proposes that the potential regulatory mechanism of this biotransformation pathway is related to the introduction of biological thiols, which strengthens the hydrogen bonding between NODR-BTPs and PP1, thereby altering the interaction between the catalytic center (Mn^2+^) of PP1 and NODR-BTPs. To better understand this mechanistic process, the key interaction sites and interactions identified through screening are illustrated in Figure 8.

A previous study by Maynes JT et al. demonstrated that motuporin (nodularin-V, a variant of nodularin) engages in extensive hydrogen-bond contacts with PP1 [31], including strong interactions with Arg_96_, Tyr_134_, Arg_221_, and Tyr_272_. These strong interactions involve conserved carboxylate-group domains located on the γ-linked glutamate and MeAsp residue carboxylates. Moreover, motuporin indirectly interacts with the active site metals via hydrogen bonds mediated by bridging water molecules. In this study, it was found that not only does the aforementioned strong interaction exist between NODR and PP1, but a hydrogen bond is also formed between NODR and Asp_220_, and an ionic bond between NODR and Ser_129_ (Figure 8A). Following the introduction of biological thiols, the most notable structural change induced by the altered steric hindrance of Mdhb^5^ residue is an increase in its surface areas, including the combination area (Figure 5A), positive accessible surface area (Figure 5B), negative accessible surface area (Figure 5C), hydrophobic surface area (Figure 5D), and polar surface area (Figure 5E). Concurrently, the positive accessible surface area and hydrophobic surface area between Adda^3^ and PP1 decreased, while Adda^3^ is closely associated with the toxicity of NODR [16]. Therefore, it is hypothesized that these surface area changes (increased Mdhb^5^ surface areas and decreased Adda^3^ surface areas) may be directly related to the reduction in NODR toxicity by the transformation products, which aligns with previous findings on the biotransformation pathways of MCLR (one of the most extensively studied microcystins). In previous studies, it was found that the introduced biological thiols directly disrupted the covalent bond between the toxin and Cys_269_ at the catalytic center of protein phosphatase 2A(PP2A), while simultaneously enhancing the hydrogen bonding interactions between Arg_268_ and the transformation products [24]. In this study, we further observed that the introduced biological thiols significantly enhanced the hydrogen bonds: “Mdhb^5^” → Glu_275_, Glu^4^ ← Arg_96_, and MeAsp^1^ ←Arg_96_ (Figure 8B). Based on previous studies by Kelker MS et al. and Maynes JT et al. on the NODR and its PP1-binding site [30,31], it is hypothesized that the enhancement of hydrogen bonds may contribute to the stabilization of the active site, while alterations in hydrogen bonding could also indirectly affect the interaction between the toxin and the active site. Our results showed that the ionic bond between Asp_92_ and Mn^2+^ was strengthened, and the metal bonds (Mn^2+^-Asp_64_, Mn^2+^-His_66_) were also enhanced, whereas the metal bond between Mn^2+^ and MeAsp^1^ was weakened (Figure 8C,D), Notably, this finding differs from previous research on MCLR biotransformation, where metal bonds between the transformation products and PP2A were uniformly enhanced [24]. This discrepancy may stem from differences in the study subjects (i.e., toxin type: NODR vs. MCLR; phosphatase type: PP1vs PP2A). Regardless, these biotransformation products influence the interaction between the toxin and the active site, thereby affecting the catalytic activity of the center. Additionally, the ionic bonds between Arg_96_-Mdhb^5^, Asp_220_-Arg^2^, and Glu_275_-Mdhb^5^ were significantly weakened (Figure 5H), which may be related to metal ion acquisition and warrants further investigation.

Previous studies on phosphatases have identified nine strictly conserved amino acids within the catalytic core region of PP1 [28]. Among them, six residues (Asp_64_, His_66_, Asp_92_, Asn_124_, His_174_, His_248_) coordinate the Mn^2+^ ions, while three residues (Arg_96_, His_125_, Arg_221_) bind the phosphate group. This study found that alterations in six conserved amino acids interacting with Mn^2+^ led to changes in the coordination structure, thereby increasing Mn^2+^ exposure. These changes primarily involved Asp_64_, His_66_, and Asp_92_, which coordinate with Mn^2+^. Additionally, altered interactions between these three conserved amino acid residues and the substrate phosphate group disrupted the normal phosphate-binding pattern. Notably, the binding of Arg_221_ to the phosphate group is significantly enhanced compared to that of NODR (Figure 8E). Combined with the aforementioned data analysis, it may be precisely the alteration in the ligand that led to changes in the interaction between the ligands and PP1, thereby modifying parameters such as the combination area.

In summary, the alteration in steric hindrance in the Mdhb^5^ residue led to an increase in ligand surface areas. The introduction of biological thiols enhanced the hydrogen bonding between NODR-BTPs and PP1, thereby affecting the stability of the PP1’s active site. This, in turn, altered the metal contact or ionic contacts among Asp_92_, Asp_64_, His_66_, and Mn^2+^, resulting in increased Mn^2+^ exposure. Consequently, the binding of the phosphate group to Arg_221_ is promoted, thereby reducing the inhibitory effect of NODR-BTPs on PP1. In this study, the biotransformation involving biological thiols primarily reduces toxin toxicity by indirectly influencing the binding at the catalytic center through the enhancement of hydrogen bonding interactions. This demonstrates that biotransformation via biological thiols constitutes an effective pathway for detoxification.

This study anchors its findings within the established knowledge framework of PP1 inhibition. Based on the structural and functional similarities between MCLR and NODR, insights drawn from prior research on the biotransformation pathways of MCLR are leveraged to refine and elaborate the current mechanistic understanding of NODR’s biotransformation pathways. Analysis of the molecular mechanisms facilitates a comprehensive assessment of the toxicity of NODR-BTPs and supports the risk regulation of their latent hazards, thereby mitigating the secondary environmental risks posed by NODR-BTPs and alleviating their adverse impacts on human health. However, this study remains exploratory in nature and lacks biological/environmental validation; therefore, the findings are limited to the in vitro level.

## 3. Conclusions

This study investigated the role of biotransformation pathways in regulating PP1 toxicity by NODR and its potential mechanisms. Six representative NODR-BTPs were prepared and purified, and their regulatory effects on biotransformation pathways were verified through PP1 inhibition experiments. Results showed that biotransformation serves as an effective strategy for modulating NODR toxicity. This study systematically investigated the role of biotransformation pathways in regulating the toxicity of Nodularin-R (NODR) toward protein phosphatase 1 (PP1) and its underlying molecular mechanisms. Six representative NODR biotransformation products (NODR-BTPs) were synthesized and purified. The regulatory effect of biotransformation pathways on protein phosphatase activity was validated through PP1 inhibition assays. Results indicate that biotransformation serves as an effective pathway for regulating the biological toxicity of NODR-BTPs, thereby attenuating NODR toxicity. Molecular simulation techniques, combined with Pearson’s correlation analysis and SPSS analysis, identified key interaction parameters and sites, suggesting a potential molecular mechanism for biotransformation pathway regulation of NODR. This study provides a molecular-level theoretical foundation for a deeper understanding of NODR toxicity regulation mechanisms and offers valuable insights for evaluating the secondary environmental risks of NODR-BTPs and formulating relevant biosafety strategies.

## 4. Materials and Methods

### 4.1. Materials

The NODR standard was acquired from Sigma (Saint-Quentin-Fallavier, France). The unit-dose reagents utilized in the experiment comprised thioacetic acid (AcSH), bovine serum albumin (BSA), cysteine-glycine (Cys-Gly), cysteine (Cys), glutathione (GSH), 2-hydroxyethyl hydrosulfide (βME), homocysteine (Hcy), manganese chloride (MnCl^2^), disodium *p*-nitrophenyl phosphate (*p*-NPP), dithiothreitol, and tris(hydroxymethyl)aminomethane (Tris), which were all sourced from Sinopharm Chemical Reagent Co., Ltd. (Shanghai, China). PP1 (1200 U/mL), sourced from rabbit skeletal muscle, was obtained from EMD Millipore (Darmstadt, Germany). Acetonitrile (ACN), trifluoroacetic acid (TFA), and methanol of HPLC grade were acquired from Merck, Darmstadt, Germany.

### 4.2. Preparation of NODR-BTPs

NODR-BTPs were synthesized by incubating 2 µM NODR with 500 µM of AcSH, Cys, Cys-Gly, GSH, βME, or Hcy in a 5% K_2_CO_3_ solution at 25 °C for 2 h. Following the reaction, the mixture was neutralized using 0.2 M HCl and subsequently applied to a pretreated Cleanert C18 solid-phase extraction (SPE) column (500 mg, Bonna-Agela Technologies, Tianjin, China). The column underwent sequential washing with 10 mL of 100% methanol, followed by 15 mL of distilled water. NODR-BTPs and impurities were fractionally eluted with 10 mL of 10% ACN and 10 mL of 80% ACN, respectively. The eluates were evaporated using a nitrogen stream, and the resulting residues were reconstituted in 1 mL of acetonitrile (ACN). The final samples were preserved at −20 °C for later analysis [32].

### 4.3. Identification of NODR-BTPs

#### 4.3.1. Preliminary Structural Identification of NODR-BTPs

The crude extract of NODR-BTPs was combined with an equal volume of ACN containing 0.1% TFA and subsequently injected into a maXis UHR-TOF high-resolution mass spectrometer for initial structural identification. Standard instrument parameters were established as follows: positive electrospray ionization (ESI+) mode; ion source voltage of 28 kV; cone voltage of 0.4 kV; desolvation gas (N_2_) pressure at 0.4 bar; dry gas (N_2_) temperature set to 180 °C; dry gas flow rate maintained at 4 L/min; full scan mass range of *m*/*z* 300,000–1,500,000. The parameters for tandem mass spectrometry were consistent with those of mass spectrometry analysis, with the exception of utilizing N_2_ as the collision gas and adjusting the collision energy to 40–50 eV.

#### 4.3.2. Purification of NODR-BTPs

The separation and purification of NODR-BTPs were conducted using a Dionex Ultimate 3000 high-performance liquid chromatography system featuring a C18 reversed-phase column. The chromatographic parameters were established as follows: Injection volume was 100 µL, with a column temperature of 35 °C and a flow rate of 2 mL/min. Mobile phase A comprised ultrapure water with 0.1% TFA, whereas mobile phase B consisted of ACN with 0.1% TFA. A linear gradient elution program was implemented as detailed below: From 0 to 5.0 min, the composition was 20% B. From 5.0 to 35.0 min, phase B increased linearly from 20% to 80%. From 35.0 to 40.0 min, the composition was maintained at 80% B. Finally, from 40.0 to 45.0 min, the composition returned to 20% B for system re-equilibration. The separated fractions were subsequently analyzed using a maXis UHR-TOF high-resolution mass spectrometer (Bruker, Beijing, China). Specific tandem mass spectrometry parameter settings are detailed in Section 4.3.1. The purified NODR-BTP fractions were collected in plastic centrifuge tubes at specific retention time windows. Following drying under a nitrogen stream, the mass of the obtained samples was determined by accurately measuring the weight difference in the centrifuge tubes before and after collection using a PL2002 electronic balance (Mettler Toledo, Shanghai, China). Finally, the dried products were redissolved in 100 or 200 µL of ACN for subsequent use.

### 4.4. The PP1 Inhibition Assay of NODR and NODR-BTPs

Using a protein phosphatase inhibition assay (primarily based on the experimental method of Zong et al. [33]), the inhibitory effect of NODR-BTPs on PP1 was evaluated by measuring PP1 activity. PP1 was diluted to a concentration of 2 U/mL in a buffer comprising 1 g/L bovine serum albumin (BSA), 1 mM MnCl_2_, 2 mM dithiothreitol, and 50 mM Tris-HCl. Subsequently, 10 µL of the PP1 solution was combined with 100 µL of the sample in a 96-well polystyrene microplate and gently agitated for 30 min. Following the mixing process, 90 µL of *p*-nitrophenyl phosphate (*p*-NPP) was added to each well, and the mixture was incubated at room temperature for 1 h. The absorbance at 405 nm (OD_405_) was quantified utilizing a Thermo/Max microplate reader. All experiments were conducted in triplicate. The inhibition rate of PP1 was determined using the following formula: I_PP1_(%) = (A_toxins_ − A_blank_)/(A_control_ − A_blank_) × 100%. In the control group, NODR-BTPs were substituted with an equivalent volume of H_2_O, whereas in the blank group, both NODR-BTPs and PP1 were replaced with H_2_O.

### 4.5. Molecular Simulations Between NODR/NODR-BTPs and PP1

The molecular modeling study was conducted with the Molecular Operating Environment (MOE, version 24.06; Chemical Computing Group, Montreal, QC, Canada). The experimental procedure was conducted as follows: First, the original crystal structure of the NODR-PP1 complex (PDB ID: *3E7A*) was retrieved from the Protein Data Bank. Following import into MOE, the NODR-PP1 complex model was processed with the “QuickPrep” module for structural correction, protonation, and removal of unbound water molecules, thus completing the structural optimization of the PP1. Subsequently, a preliminary interaction model between NODR-BTPs and PP1 was constructed using a ligand replacement strategy: the ligand NODR in the NODR-PP1 model was substituted with NODR-BTPs. The NODR-BTP and PP1 model was protonated again using the “QuickPrep” module to complete ligand structure optimization. Subsequently, the model underwent energy minimization via “Minimiz” to enhance structural stability. In MOE software (version 24.06), the default method for generating conformations is the Rotate Bonds. Next, the ligands, NODR/NODR-BTPs, were docked into the PP1 binding site (the catalytic center of PP1: the two manganese ions) using a “General Docking” mode [34]. In this mode, the Triangle Matcher method was used to generate poses, which aligns the three-atom groups of the ligand with the three-atom groups of the alpha spheres in a more systematic manner, thereby enabling the generation of poses in a more systematic way and ensuring better docking between PP1 and the toxins. The force field parameters used were Amber 19 EHT, an all-atom force field combining EHT and Amber19. This force field is compatible with RESP and AM1-BCC charges. The pH was set to 7.5, the temperature to 25 °C, and the salinity to 0.05 M. This mode generated up to 30 poses per ligand. The scoring function used was London dG; the London dG scoring function estimates the free energy of binding of the ligand from a given pose.The resulting poses were sorted by a scoring function—more negative values indicate higher predicted binding affinity. Finally, the candidate interaction parameters relevant to the binding of NODR/NODR-BTPs with PP1 were obtained, including the following: negative accessible surface area; positive accessible surface area; combination area; polar surface area; exposure area associated with phosphate group; hydrophobic surface area; active center exposure; ionic bonds; metal bonds; hydrogen bonds. We selected the average of the candidate parameters for the five lowest-scoring groups of poses among the 30 poses.

### 4.6. Statistical Analysis

The results from the PP inhibition assays were presented as the mean ± standard error (*n* = 3). The relationship between inhibition data and pertinent parameters was examined utilizing IBM SPSS Statistics (version 27.0; IBM Corp., Chicago, IL, USA). Statistical significance was categorized into three levels: highly significant (*p* < 0.01), significant (*p* ≤ 0.05), and non-significant (*p >* 0.05). Differences between the NODR and NODR-BTP groups were assessed using one-way analysis of variance (ANOVA), followed by the least significant difference (LSD) post hoc test. A *p*-value of less than 0.05 was deemed statistically significant.

## Data Availability

All data supporting this study are included in the article and its Supplementary Materials. Further inquiries can be directed to the corresponding author.

## Supplementary Materials

The following supporting information can be downloaded at https://www.mdpi.com/article/10.3390/toxins18020091/s1, Figure S1: MS spectra for NODR (A), NODR-βME (B), NODR-AcSH (C), NODR-Cys (D), NODR-Hcy (E), NODR-GSH (F), NODR-Cys-Gly (G); Figure S2: MS/MS spectra for NODR (A), NODR-βME (B), NODR-AcSH (C), NODR-Cys (D), NODR-Hcy (E), NODR-GSH (F), NODR-Cys-Gly (G); Table S1: MS/MS identification of NODR and NODR-BTPs; Table S2: Preparation and purification information for the electrophilic addition samples of NODR; Table S3: The candidate interaction parameters between NODR/NODR-BTPs and PP1; Table S4: Pearson correlation analysis of inhibition data and the candidate interaction parameters.
