# Supplementary material for: Biotransformation Is an Effective Mechanism for Modulating the Biological Toxicity of Nodularin (NODR)
Source: Toxins (Basel). 2026 Feb 11;18(2):91. doi: 10.3390/toxins18020091 (PMC12944905; doi:10.3390/toxins18020091)
Supplement: Supplementary file 1 [file toxins-18-00091-s001.zip › toxins-4107409-supplementary.pdf]

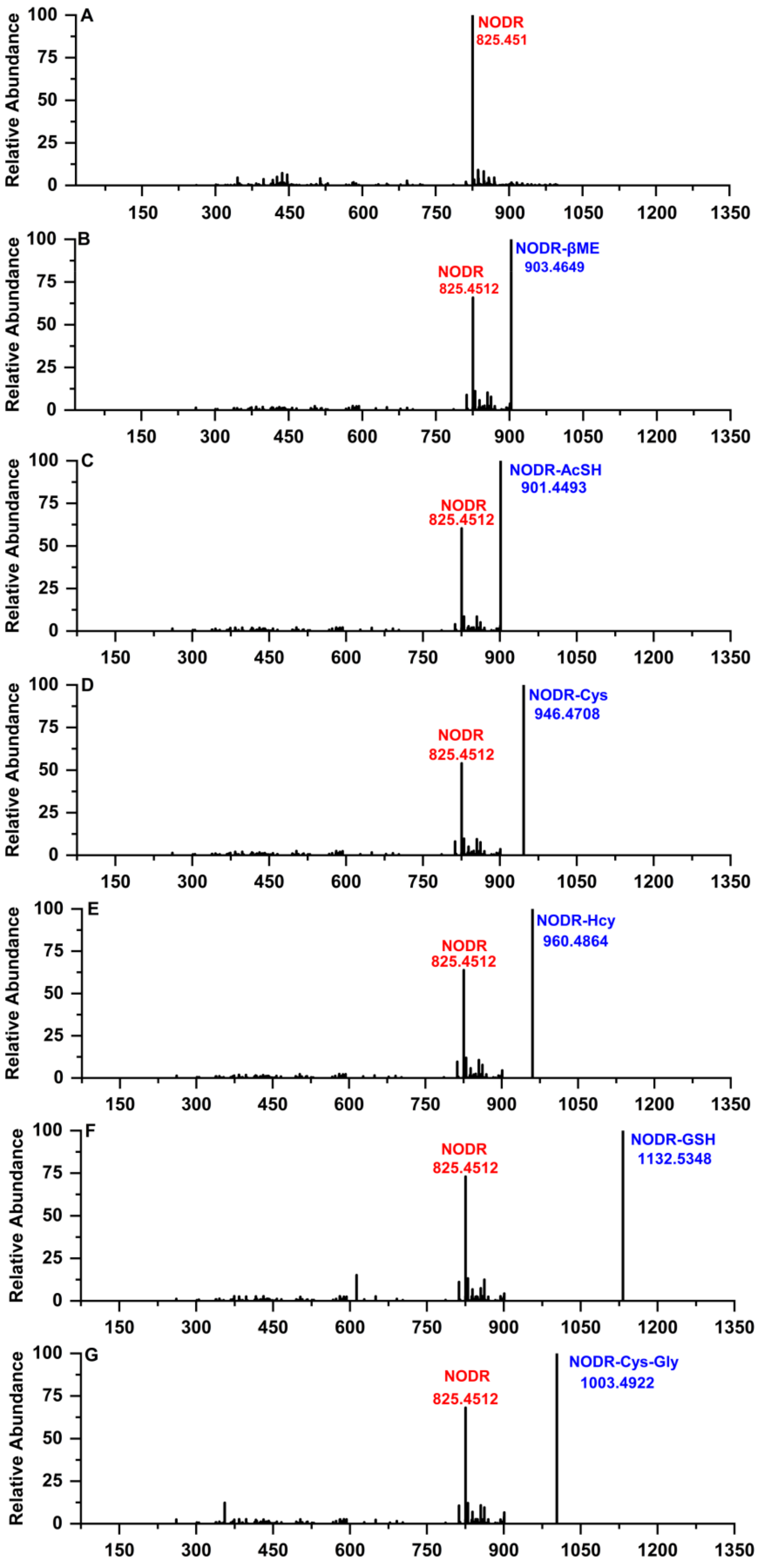

Figure S1. MS spectra for NODR (A), NODR-βME (B), NODR-AcSH (C), NODR-Cys (D), NODR-Hcy (E), NODR-GSH (F), NODR-Cys-Gly (G)

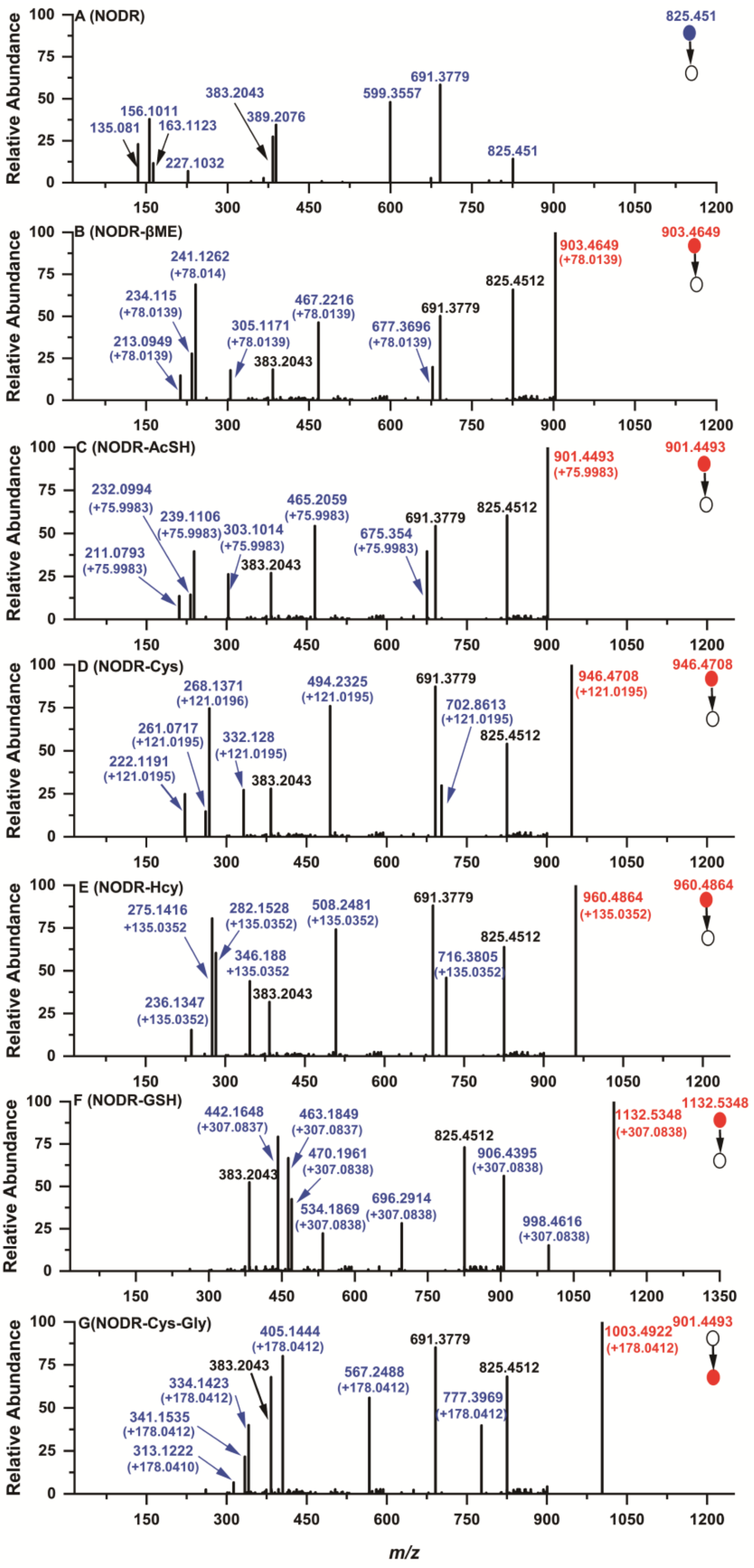

Figure S2. MS/MS spectra for NODR (A), NODR-βME (B), NODR-AcSH (C), NODR-Cys (D), NODR-Hcy (E), NODR-GSH (F), NODR-Cys-Gly (G)

Table S1 MS/MS identification of NODR and NODR-BTPs

Basic fragment ions	NODR	NODR-βME	NODR-AcSH	NODR-Cys	NODR-Hcy	NODR-GSH	NODR-Cys-Gly
$[M+H]^+$	825.451	903.4649	901.4493	946.4708	960.4864	1132.5348	1003.4922
		(+ 78.0139) ↑[a]	(+ 75.9983) ↑	(+ 121.0197) ↑	(+ 135.0354) ↑	(+ 307.0838) ↑	(+ 178.0412) ↑
[-Glu[4]-Mdhb[5]-MeAsp[1]-Arg[2]-$C_{11}H_{17}NO$-]+2H]$^+$	691.3779	691.3779√[b]	691.3779√	691.3779√	691.3779√	691.3779√	691.3779√
[$C_{11}H_{15}O$-Glu[4]-Mdhb[5]-]$^+$	389.2076	467.2216↑	465.2059↑	510.2274↑	524.243↑	696.2914↑	567.2488↑
[-$C_{11}H_{15}O$-]$^+$	163.1123	241.1262↑	239.1106↑	284.132↑	298.1477↑	470.1961↑	341.1535↑
[(Arg[2]-Adda[3]-Glu[4])+H]$^+$	599.3557	677.3696↑	675.354↑	720.3754↑	732.3754↑	906.4395↑	777.3969↑
[(Mdhb[5]-MeAsp[1]-Arg[2])+H]$^+$	383.2043	383.2043√	383.2043√	383.2043√	383.2043√	383.2043√	383.2043√
[(- Glu [4]-Mdhb[5])+H]$^+$	227.1032	305.1171↑	303.1014↑	348.1229↑	362.1386↑	534.1869↑	405.1444↑
[(-Arg[2]-)+H]$^+$	156.1011	234.115↑	232.0994↑	277.1208↑	291.1365↑	463.1849↑	334.1423↑
[PhCH2CH(OCH3)]$^+$	135.081	213.0949↑	211.0793↑	256.1007↑	270.1164↑	442.1648↑	313.1222↑
Target residue	-			Mdhb[5]			

a:↑and↓mean mass changes were related to these fragment ions; **b:**√means ions with the stable m/z were detected by mass spectrograph.

Table S2 Preparation and purification information for the electrophilic addition samples of NODR

**Conjugation products**	**Eluted time**[a]	**Concentration**[b]	**Total volume**	**Purity**[C]
NODR-βME	19.7min	≈1200 μmol/L	5x100μL	98.3%
NODR-AcSH	20.1 min	≈1000 μmol/L	5x100μL	97.2%
NODR-Cys	17.4min	≈1100 μmol/L	5x100μL	98.9%
NODR-Hcy	17.3min	≈1100 μmol/L	5x100μL	98.0%
NODR-GSH	12.5min	≈1200 μmol/L	5x100μL	98.5%
NODR-Cys-Gly	15.8min	≈1200 μmol/L	$5 \times 100\mu L$	96.7%

[a] Collection time was set for 0.5 min (± 0.25 min around the eluted time).

[b] With 200 μmol/L NODR served as the inner standard for quantification and assumed NODR and NODR-BTPs had approximate protonated efficiencies.

[c] Purity was directed calculated by the MS signals of NODR and NODR-BTPs.

Table S3. The candidate interaction parameters between NODR/NODR-BTPs and PP1

Molecular simulation parameters		NODR	NODR-βME	NODR-Acsh	NODR-Cys	NODR-Hcy	NODR-GSH	NODR-Cys-Gly
Combination area(Å^2)	Total	568.7453	637.8885	652.5443	660.3479	563.1901	661.2222	643.5648
	MeAsp^1→PP1	56.9673	63.1679	63.3224	65.1688	63.7585	78.4371	59.3246
	Arg^2→PP1	117.2544	128.3549	122.4422	117.5843	126.1884	116.0503	103.6700
	Adda^3→PP1	312.45300	356.9385	368.7243	359.1755	319.6869	311.8917	336.8714
	Glu^4→PP1	164.9017	172.4435	173.3049	173.9342	171.4528	172.6258	147.9794
	Mdhb^5→PP1	137.37445	161.21608	161.89648	176.66379	178.12499	238.53226	191.96601
Positive accessible surface area(Å^2)	Total	325.0009	364.2941	365.8074	380.7407	367.7018	387.5286	364.8284
	MeAsp^1→PP1	30.7547	35.8743	36.8097	36.0388	36.9895	35.3446	28.4002
	Arg^2→PP1	74.0749	82.8533	76.7166	76.8949	86.4326	76.1222	64.7171
	Adda^3→PP1	189.7979	212.0701	222.7961	220.5245	195.4990	196.4035	205.5987
	Glu^4→PP1	94.5194	100.9941	102.3349	102.7616	102.9558	102.7398	86.8873
	Mdhb^5→PP1	86.9484	105.1828	101.3289	110.4239	122.7772	150.1455	115.9552
Negative accessible surface area(Å^2)	Total	243.7444	273.5944	286.7370	279.6069	249.4882	220.9519	278.59555
	MeAsp^1→PP1	26.7128	27.8025	27.0135	29.6297	27.1690	73.3423	31.4244
	Arg^2→PP1	44.1804	46.5016	46.7256	41.6892	40.6560	45.6336	39.7117
	Adda^3→PP1	124.1552	146.3684	147.4282	140.1508	125.6879	105.5611	132.6312
	Glu^4→PP1	73.3558	73.4492	72.9696	83.1723	70.4972	78.9489	63.0928
	Mdhb^5→PP1	47.6523	58.5332	63.0674	68.7406	57.8478	79.8269	78.5106
Hydrophobic surface area(Å^2)	Total	321.1081	353.1629	365.4495	331.8195	332.6250	334.6309	327.2204
	MeAsp^1→PP1	3.2199	1.9233	2.6006	3.6676	2.9045	14.1281	4.8835
	Arg^2→PP1	42.1718	46.3338	41.4964	41.6887	49.2543	36.2868	34.9287
	Adda^3→PP1	196.5138	237.1244	244.1284	155.7073	198.7388	198.1335	215.1593

	Glu^4→PP1	50.7805	51.4534	53.0767	51.3962	53.4300	56.8588	41.2342
	$Mdhb^5$→PP1	92.8358	81.9338	91.0689	65.1709	95.6716	110.2025	79.3527
Polar surface area(Å^2)	Total	247.6372	284.7256	287.0947	328.5282	284.5652	326.5912	312.3442
	$MeAsp^1$→PP1	54.2475	61.7445	61.2214	62.0010	61.3542	58.8091	54.9410
	Arg^2→PP1	76.0828	83.0210	81.9458	76.8956	77.9344	80.7637	59.6408
	$Adda^3$→PP1	117.4395	121.3147	126.0955	128.6585	122.4483	115.2581	123.2117
	Glu^4→PP1	117.0948	122.9901	122.2275	114.5378	120.0234	117.7669	89.6459
	$Mdhb^5$→PP1	44.2084	81.7824	73.3272	113.9937	84.9537	130.8299	115.1132
Active center exposure(Å^2)	Mn_1^{2+}+Asp_{64}	321.6888	317.6289	317.1947	317.3350	317.0767	314.4149	317.6207
	Mn_1^{2+}+His_{66}	350.92144	350.0905	349.4710	351.5541	348.9258	351.1042	349.8822
	Mn_1^{2+}+Asp_{92}	319.0623	317.0431	316.1286	313.9695	314.3112	316.9431	317.1865
	Mn_1^{2+}＋Asp_{64}＋His_{66}+Asp_{92}	571.61458	570.9214	566.9012	572.1340	561.2146	574.4534	572.7901
	Mn_2^{2+}+Asp_{64}	323.03286	320.8804	321.1939	323.0950	320.2053	317.7085	324.3702
	Mn_2^{2+}+Asp_{92}	309.6006	309.7095	308.8937	306.9446	308.0485	309.1487	309.5152
	Mn_2^{2+}+Asn_{124}	323.1800	322.3894	322.9242	322.6823	319.9037	324.7952	322.8160
	Mn_2^{2+}+His_{248}	340.8752	340.5209	341.3278	341.5457	338.8298	344.7952	341.8221
Exposure area associated with -PO_4(Å^2)	Arg_{96} + His_{125} + Arg_{221}	1012.7824	1027.3282	1018.0926	1044.1173	1011.2006	1030.6948	1028.97
	Arg_{96}	375.0971	373.937	374.2575	376.0859	372.5666	377.0253	376.2827
	His_{125}	318.2926	318.025	320.4839	320.2924	318.6196	319.5115	318.6328
	Arg_{221}	375.0361	374.2714	373.1814	373.4975	373.1437	371.7457	371.0806
Hydrogen bond (KJ/Mol)	Total	-13.7	-31.5	-27.1	-28.5	-28.1	-58.8	-29.3
	Arg_{96}→$MeAsp^1$	-0.5	-3.3	-3.1	-5.4	-3.7	-5	-3.3
	Arg_{96}→Glu^4	-0.7	-3.4	-5.3	-6.2	-7.6	-8	-4.7

	Arg$_{96}$→Mdhb^{5}	-	-1.6	-0.7	-0.6	-0.9	-1.6	-
	Asn$_{124}$→Adda^{3}	-3.7	-3.1	-3	-0.9	-3.3	-1.5	-3.3
	His$_{125}$→Adda^{3}	-2	-0.6	-1.6	-1.3	-1.5	-1.9	-1.5
	Tyr$_{134}$→MeAsp^{1}	-0.6	-4	-3.9	-2.3	-3.4	-	-
	Arg$_{221}$→Arg^{2}	-2.8	-5.6	-3.4	-1.5	-4.6	-0.5	-4.5
	Tyr$_{272}$→Glu^{4}	-3.4	-4.4	-3.4	-5.2	-4.5	-5.2	-3.5
	Cys$_{273}$←Mdhb^{5}	-0.3	-0.5	-	-	-	-2.3	-1.4
	Glu$_{275}$→Mdhb^{5}	-	-	-	-3.7	-	-0.7	-
	Glu$_{275}$←Mdhb^{5}	-	-3.2	-4.8	-	-12.4	-28.9	-14.3
Metal bond （KJ/Mol）	Total	-35.5	-37	-37	-36.3	-36.2	-38.1	-33.3
	Mn$_{1}^{2+}$-MeAsp^{1}	-4.2	-1.5	-	-	-	-	-
	Mn $_{1}^{2+}$-Glu^{4}	-	-1.6	-3.4	-3.5	-3.4	-3.4	-1.7
	Mn $_{1}^{2+}$-Asp$_{64}$	-5.2	-5.3	-5.4	-5.5	-5.4	-5.4	-5.5
	Mn $_{1}^{2+}$-His$_{66}$	--	-	-0.8	-0.9	-1.1	-3.5	-
	Mn $_{1}^{2+}$-Asp$_{92}$	-4.7	-5	-4.9	-4.9	-5	-4.9	-5
	Mn $_{2}^{2+}$-Asp$_{64}$	-5.1	-5	-5	-5	-5	-5.1	-4.9
	Mn $_{2}^{2+}$-Asp$_{92}$	-6.7	-6.7	-6.6	-7	-6.6	-6.9	-6.7
	Mn$_{2}^{2+}$-Asn$_{124}$	-3.8	-3.8	-3.6	-3.9	-3.9	-4.1	-3.8
	Mn $_{2}^{2+}$-Hiss$_{248}$	-5.8	-5.7	-5.8	-5.6	-5.8	-4.8	-5.7
Ionic bond （KJ/Mol）	Total	-72.93	-76.4	-79.3	-94.6	-80.5	-80.4	-71.8
	Arg$_{96}$-MeAsp^{1}	-3.6	-2.9	-6.7	-7.7	-7.2	-6.2	-
	Arg$_{96}$-Glu^{4}	-3.73	-7.3	-7.1	-5.6	-6.6	-7.1	-6.1
	Arg$_{96}$ -Mdhb^{5}	-	-	-8.3	-6	-	-	-
	Asp$_{220}$-Arg^{2}	-	-2.1	-	-1.2	-	-	-
	Glu$_{275}$-Mdhb^{5}	-	-	-	-5.1	-	-3.2	-

	Mn_1^{2+}-Glu[4]	-10.3	-7.5	-8.8	-9.4	-9.7	-9.9	-6.7
	Mn_1^{2+}-Asp_{64}	-11.3	-12	-12.2	-14.7	-12.3	-12.5	-14.4
	Mn_1^{2+}-Asp_{92}	-11	-11.9	-11.9	-12	-12.1	-11.8	-11.9
	Mn_2^{2+}-Asp_{64}	-11.3	-11.1	-11.1	-11.1	-11.2	-11.3	-11
	Mn_2^{2+}-Asp_{92}	-21.7	-21.6	-21.5	-21.8	-21.4	-21.6	-21.7

[a:] - no related parameter was detected

Table S4 Pearson correlation analysis of inhibition data and the candidate interaction parameters

Pearson Correlation Analysis Data		Combination area（Å^2）					
		Total	MeAsp^1→PP1A	Arg^2→PP1A	Adda^3→PP1A	Glu^4→PP1A	“Mdhb^5”→PP1A
1 nM	R^a(P^b)	-0.399(0.187)	-0.598(0.078)	0.350(0.221)	0.106(0.411)	0.115(0.403)	-0.852**(0.007)
10 nM	R (*P*)	-0.360(0.214)	-0.576(0.088)	0.438(0.163)	0.206(0.329)	0.209(0.327)	-0.869**(.006)
100nM	R (*P*)	-0.327(0.237)	-0.573(0.089)	0.568(0.092)	0.350(0.221)	0.317(0.244)	-0.895**(0.003)
Pearson Correlation Analysis Data		Positive accessible surface area（Å^2）					
		Total	MeAsp^1→PP1A	Arg^2→PP1A	Adda^3→PP1A	Glu^4→PP1A	“Mdhb^5”→PP1A
1 nM	R (*P*)	-0.840**(0.09)	-0.133(0.388)	0.101(0.414)	-0.065(0.445)	-0.088(0.425)	-0.850**(0.008)
10 nM	R (*P*)	-0.781*(0.019)	-0.015(0.487)	0.179(0.351)	0.037(0.468)	0.012(0.490)	-0.856**(0.007)
100nM	R (*P*)	-0.676*(0.048)	0.145(0.379)	0.316(0.245)	0.178(0.351)	0.139(0.383)	-0.857**(0.007)
Pearson Correlation Analysis Data		Negative accessible surface area（Å^2）					
		Total	MeAsp^1→PP1A	Arg^2→PP1A	Adda^3→PP1A	Glu^4→PP1A	“Mdhb^5”→PP1A
1 nM	R (*P*)	0.128 (0.392)	-0.514 (0.119)	0.480 (0.138)	0.363 (0.212)	-0.013 (0.489)	-0.836**(0.010)
10 nM	R (*P*)	0.196 (0.337)	-0.547 (0.102)	0.530 (0.111)	0.444 (0.159)	0.027 (0.477)	-0.848**(0.008)
100nM	R (*P*)	0.315 (0.245)	-0.634 (0.063)	0.518 (0.117)	0.580 (0.086)	0.071 (0.440)	-0.862**(0.006)
Pearson Correlation Analysis Data		Hydrophobic surface area（Å^2）					
		Total	MeAsp^1→PP1A	Arg^2→PP1A	Adda^3→PP1A	Glu^4→PP1A	“Mdhb^5”→PP1A
1 nM	R (*P*)	0.142 (0.380)	-0.550 (0.101)	0.347 (0.223)	0.302 (0.255)	0.022 (0.481)	-0.075 (0.436)
10 nM	R (*P*)	0.259 (0.287)	-0.595 (0.080)	0.409 (0.181)	0.342 (0.227)	0.084 (0.429)	-0.093 (0.421)
100nM	R (*P*)	0.376 (0.203)	-0.697*(0.041)	0.544 (0.104)	0.350 (0.221)	0.125 (0.395)	-0.189 (0.342)
Pearson Correlation Analysis Data		Polar surface area（Å^2）					
		Total	MeAsp^1→PP1A	Arg^2→PP1A	Adda^3→PP1A	Glu^4→PP1A	“Mdhb^5”→PP1A
1 nM	R (*P*)	-0.852**(0.008)	-0.221 (0.317)	0.304 (0.253)	-0.136 (0.385)	0.390 (0.194)	-0.900**(0.003)
10 nM	R (*P*)	-0.834**(0.010)	-0.099 (0.417)	0.387 (0.195)	-0.049 (0.458)	0.469 (0.144)	-0.907**(0.002)

100nM	R (*P*)	-0.781*(0.019)	0.099 (0.416)	0.465 (0.147)	0.465 (0.409)	0.546 (0.102)	-0.883**(0.004)
Pearson Correlation Analysis Data		Hydrogen bond (KJ/Mol)）					
		Total	Arg96→ MeAsp1	Arg96→Glu4	Arg96→"Mdhb^{5}"	ASN124→ Adda3	HIS125→Adda3
1 nM	R (*P*)	0.676*(0.048)	0.818*(0.012)	0.893*(0.003)	0.234 (0.307)	-0.53 (0.110)	0.061(0.448)
10 nM	R (*P*)	0.672*(0.049)	0.765*(0.023)	0.836**(0.009)	0.18 (0.350)	-0.51(0.126)	0.113(0.405)
100nM	R (*P*)	0.683*(0.046)	0.653 (0.056)	0.742* (0.028)	0.099 (0.416)	-0.452(0.155)	0.263(0.285)
Pearson Correlation Analysis Data		Hydrogen bond (KJ/Mol)					
		TYR134→ MeAsp^{1}	ARG221→Arg^{2}	TYR272→Glu^{4}	CYS273←"Mdhb^{5}"	GLU275← "Mdhb^{5}"	GLU275→ "Mdhb^{5}"
1 nM	R (*P*)	-0.244 (0.299)	-0.277 (0.274)	0.551(0.1)	0.931*(0.35)	0.871*(0.027)	0.290 (0.264)
10 nM	R (*P*)	-0.364(0.211)	-0.293(0.262)	0.531 (0.110)	0.931*(0.034)	0.870*(0.028)	0.267(0.281)
100nM	R (*P*)	-0.549(0.101)	-0.37 (0.207)	0.456(0.152)	0.941*(0.029)	0.893*(0.021)	0.197(0.336)
Pearson Correlation Analysis Data		Metal bond （KJ/Mol）					
		Total	MN $_{1}^{2+}$- MeAsp^{1}	MN $_{1}^{2+}$-Glu^{4}	MN $_{1}^{2+}$-ASP 64	MN $_{1}^{2+}$-HIS66	MN $_{1}^{2+}$-ASP92
1 nM	R (*P*)	-0.010 (0.491)	-1.00** (0.012)	0.349(0.249)	0.839**(0.009)	0.783(0.059)	0.635(0.063)
10 nM	R (*P*)	-0.131(0.390)	-1.00** (0.014)	0.226 (0.333)	0.810*(0.014)	0.802(0.051)	0.574(0.089)
100nM	R (*P*)	-0.187(0.344)	-1.00** (0.017)	0.159(0.381)	0.723*(0.033)	0.893*(0.021)	0.436(0.164)
Pearson Correlation Analysis Data		Metal bond （KJ/Mol）					
		MN$_{2}^{2+}$-ASP 64	MN $_{2}^{2+}$-ASP 92	MN$_{2}^{2+}$-ASN124	MN $_{2}^{2+}$-HIS248		

1 nM	R (*P*)	-0.338(0.229)	0.338 (0.230)	0.544(0.103)	-0.54 (0.106)		
10 nM	R (*P*)	-0.323 (0.24)	0.379 (0.201)	0.614(0.071)	-0.511 (0.100)		
100nM	R (*P*)	-0.230 (0.310)	0.393 (0.192)	0.660(0.054)	-0.622 (0.068)		
Pearson Correlation Analysis Data		Ionic bond （KJ/Mol）					
		Total	Arg96-MeAsp^{1}	Arg 96-Glu^{4}	Arg96 -“Mdhb^{5}”	ASP220-Arg2	GLU275-“Mdhb^{5}”
1 nM	R (*P*)	0.342 (0.226)	0.750* (0.043)	0.541(0.105)	-1**(0.008)	-1.00**(0.03)	-1.00**(0.007)
10 nM	R (*P*)	0.287(0.267)	0.719(0.054)	0.414(0.178)	-1**(0.007)	-1.00**(0.06)	-1.00**(0.005)
100nM	R (*P*)	0.161(0.365)	0.644 (0.084)	0.295(0.261)	-1**(0.009)	-1.00**(0.05)	-1.00**(0.003)
Pearson Correlation Analysis Data		Ionic bond （KJ/Mol）					
		MN $_{1}^{2+}$-Glu^{4}	MN $_{1}^{2+}$ -ASP64	MN $_{1}^{2+}$ -ASP 92	MN_2^{2+}-ASP64	MN_2^{2+}-ASP92	
1 nM	R (*P*)	-0.151 (0.373)	0.617 (0.070)	0.738*(0.029)	-0.211 (0.325)	-0.137(0.385)	
10 nM	R (*P*)	-0.143(0.38)	0.646(0.058)	0.641(0.060)	-0.171(0.357)	-0.040(0.466)	
100nM	R (*P*)	-0.102(0.414)	0.593 (0.080)	0.469(0.144)	-0.066(0.444)	0.035(0.47)	
Pearson Correlation Analysis Data		Active center exposure(Å^{2})					
		Mn_1^{2+}+Asp64	Mn_1^{2+}+His66	Mn_1^{2+}+Asp92	Mn_1^{2+}＋Asp64＋His66+Asp92	Mn_2^{2+}+Asp64	Mn_2^{2+}+Asp92
1 nM	R (*P*)	0.849**(0.008)	0.077(0.435)	0.571(0.09)	0.03 (0.498)	0.319(0.243)	0.393(0.192)
10 nM	R (*P*)	0.769*(0.022)	0.007(0.494)	0.492(0.131)	-0.074(0.437)	0.243 (0.300)	0.355(0.217)
100nM	R (*P*)	0.696*(0.041)	-0.075(0.436)	0.311(0.249)	-0.199(0.335)	0.224 (0.315)	0.225(0.314)
Pearson Correlation Analysis Data		Active center exposure（Å^{2}）			Exposure area associated with -PO_4(Å^{2})		
		Mn_2^{2+}+Asn124	Mn_2^{2+}+His248	Mn_2^{2+}+Asp64+Asp92 +Asn124+His248	Arg_{96} + His_{125} + Arg_{221}	Arg_{96}	His_{125}
1 nM	R (*P*)	0.001 (0.499)	-0.382 (0.199)	-0.113 (0.405)	-0.438(0.163)	-0.326(0.238)	-0.333(0.233)
10 nM	R (*P*)	-0.022(0.481)	-0.399 (0.188)	-0.195 (0.337)	-0.436(0.164)	-0.394(0.191)	-0.247(0.296)

100nM	R (*P*)	-0.157(0.368)	-0.509 (0.122)	-0.349 (0.221)	-0.395 (0.190)	-0.516(0.118)	-0.184(0.347)
Pearson Correlation Analysis Data		Exposure area associated with $-PO_4$(Å^2)					
		Arg_{221}					
1 nM	R (*P*)	0.895**(0.07)					
10 nM	R (*P*)	0.876**(0.05)					
100nM	R (*P*)	0.898**(0.03)					

[a:] R denotes the Pearson correlation coefficient between candidate interaction parameters and toxin toxicity at different toxin levels; [b:] *P* indicates the significance level of the correlation data; ** denotes significance at the 0.01 level; * denotes significance at the 0.05 level.
